# Real‐Time Predictive Analysis of ICU Ventilator Weaning Failure: A Prospective Validation Study

**DOI:** 10.1111/crj.70136

**Published:** 2025-11-04

**Authors:** Lili Zhou, Peng Zhou, Changling Gao, Taozi Li, Qiaoyun Zhou

**Affiliations:** ^1^ Department of Critical Care Medicine, South District The First Affiliated Hospital of Anhui Medical University Hefei China

**Keywords:** APACHE II score, Glasgow Coma Scale, ICU, predictive model, ventilator weaning failure

## Abstract

**Objective:**

To construct and validate a nomogram model for predicting the risk of ICU ventilator weaning failure (reintubation or death within 48 h after extubation) based on multidimensional clinical indicators.

**Methods:**

A total of 485 patients in the ICU who needed ventilator weaning were selected and divided into a training set (*n* = 340) and a validation set (*n* = 145) at a ratio of 7:3. Baseline data and weaning‐related indicators of the patients were collected. Weaning failure (reintubation or death within 48 h after weaning) was regarded as the outcome event. Independent risk factors were screened through univariate and multivariate logistic regression. A nomogram model was constructed, and the model's performance was evaluated using the C‐index, AUC, calibration curve, Hosmer–Lemeshow test, and decision curve.

**Results:**

The weaning failure rate in the training set was 28.53% (97/340), and that in the validation set was 29.66% (43/145). Multivariate regression showed that age, APACHE II score, duration of mechanical ventilation, spontaneous breathing frequency, Glasgow Coma Scale score, and the use of sedatives were independent influencing factors (*p* < 0.05). The C‐index of the nomogram model in the training set and the validation set was 0.829 and 0.826, respectively. The AUC was 0.828 (95% CI: 0.762–0.893) and 0.823 (95% CI: 0.732–0.915), respectively. The sensitivity and specificity were 0.811, 0.751 and 0.662, 0.702, respectively. The calibration curve and Hosmer–Lemeshow test (*p* = 0.109, 0.402) showed that the model had a good fit.

**Conclusion:**

The nomogram model constructed based on the above indicators can effectively predict the risk of ICU ventilator weaning failure and provide a basis for formulating individualized weaning strategies.

## Graphical abstract

This study developed and validated a nomogram model to predict ICU ventilator weaning failure using age, APACHE II, ventilation duration, and other key clinical factors. The model showed high accuracy (AUC > 0.82) and good calibration, aiding individualized weaning decisions. Results support its utility in reducing reintubation risks and improving ICU outcomes.

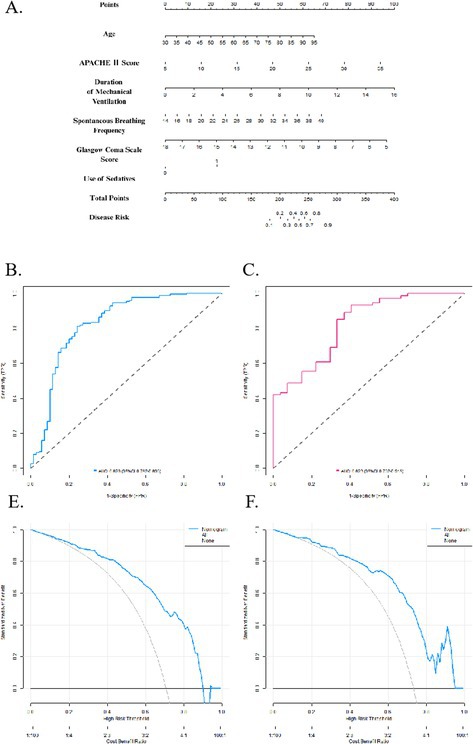

## 1 Introduction

Ventilator weaning is a crucial step for ICU patients to be disengaged from life support and achieve recovery. Its success or failure directly affects the length of hospital stay, medical costs, and prognosis [1, 2].

According to data from multiple multicenter studies, the global failure rate of ventilator weaning in ICU patients is as high as 20%–35%. In tertiary hospitals in China, the weaning failure rate is approximately 28.5%, significantly higher than the 18%–22% in European and American countries. Weaning failure not only prolongs the mechanical ventilation time of patients by 3–5 days and increases the risk of nosocomial infection by 2.3 times but also raises the mortality rate by 40%–60% compared with those with successful weaning. Among them, the 90‐day mortality rate of patients who need reintubation due to respiratory failure can reach 38.7% [3].

Currently, the clinical assessment of weaning feasibility mainly relies on single indicators such as the spontaneous breathing trial (SBT) and the oxygenation index (PaO_2_/FIO_2_). However, the sensitivity of these indicators is only 60%–70%, often resulting in “false‐negative” results. Approximately 15% of patients who pass the SBT still fail to be weaned within 48 h [4]. Although factors such as APACHE II score and duration of mechanical ventilation have been proven to be related to weaning outcomes, most existing studies focus on single‐factor analysis and lack integrated analysis of multidimensional variables such as age, state of consciousness, and treatment interventions (e.g., use of sedatives) [5, 6]. In addition, the lack of real‐time dynamic prediction tools makes it difficult for clinicians to dynamically adjust strategies during the weaning process, and they often intervene passively after failure [7].

Real‐time dynamic monitoring is critical for improving weaning success, as the physiological state of ICU patients is highly volatile. Traditional static assessments, such as a single SBT result, only capture a snapshot of the patient's condition at a specific time point and fail to reflect rapid changes in respiratory function, consciousness, or systemic stability. For example, a patient who passes the SBT in the morning may develop respiratory muscle fatigue or neurological deterioration by afternoon, which would be missed by static indicators. Real‐time prediction tools can continuously integrate multidimensional data (e.g., spontaneous breathing frequency, Glasgow Coma Scale [GCS] score) at multiple time points, enabling clinicians to identify early warning signs of weaning failure and adjust strategies proactively—such as delaying extubation or strengthening respiratory support—rather than waiting for adverse events to occur. This dynamic adjustment not only reduces the risk of reintubation but also shortens unnecessary mechanical ventilation time, thereby lowering the incidence of ventilator‐associated complications [8, 9].

In recent years, the application of prediction models in the field of critical care has provided new ideas for solving this problem. However, there are still limitations in the research on their use in ventilator weaning: Some models only include respiratory mechanics parameters, ignoring the influence of neurological function status on respiratory regulation; some models have not undergone strict external validation, and their clinical applicability is questionable [10]. In this study, based on a prospective cohort, a nomogram model for real‐time prediction of weaning failure was constructed by integrating patients' baseline characteristics, disease severity scores, respiratory parameters, and treatment measures. The aim was to improve the accuracy of weaning assessment, provide a quantitative basis for formulating individualized weaning plans in clinical practice, and ultimately reduce the failure rate and improve patients' prognosis.

## 2 Methods

### 2.1 Study Subjects

A total of 485 patients who needed ventilator weaning and were admitted to the ICU of the hospital from January 2024 to June 2025 were selected, with 140 weaning failure events (97 in the training set, 43 in the validation set). Study subjects were divided into a training set (*n* = 340) and a validation set (*n* = 145) at a ratio of 7:3. The final model contained six independent risk factors, and the number of events per variable (EPV) was 140/6 ≈ 23.3, which was much higher than the recommended minimum EPV (10), confirming the sample size was sufficient to avoid overfitting. Inclusion criteria were as follows: (1) The duration of mechanical ventilation was ≥ 24 h, (2) the patients were planned to undergo the first weaning attempt, and (3) the patients were ≥ 18‐years old. Baseline was defined as the time when patients met the weaning preparation criteria (e.g., stable hemodynamics, PaO_2_/FIO_2_ > 150 mmHg). Exclusion criteria were as follows: (1) patients with irreversible respiratory failure (defined as chronic respiratory failure with PaCO_2_ > 80 mmHg for more than 6 months) who required long‐term mechanical ventilation, (2) patients with severe craniocerebral injury or impaired consciousness who could not cooperate with the assessment, (3) patients with end‐stage organ failure before enrollment. Internal validation was performed using tenfold repeated cross‐validation (1000 iterations) combined with bootstrap sampling (1000 repetitions). For temporal validation, patients were divided into an early cohort (January 2024 to September 2024, *n* = 268) and a late cohort (October 2024 to June 2025, *n* = 217), with the early cohort as the training set and the late cohort as the independent validation set.

### 2.2 Data Collection

Patients' baseline data (measured within 24 h before the first weaning attempt) were collected, including age, gender, underlying diseases (such as chronic obstructive pulmonary disease and heart failure), laboratory indicators (such as platelet count, serum creatinine, and hemoglobin), respiratory parameters (such as spontaneous respiratory rate, tidal volume, peak airway pressure, and positive end‐expiratory pressure), scoring scales (APACHE II score and GCS score), and treatment‐related indicators (duration of mechanical ventilation, duration of SBT, and the use of sedatives). The missing rate of all variables was < 5% (e.g., platelet count: 2.1%, serum creatinine: 1.7%, and spontaneous breathing rate: 0.8%). Weaning failure was defined as reintubation within 48 h after weaning or death due to respiratory failure. All data collection procedures were conducted in accordance with the ethical guidelines approved by the Ethics Committee of the hospital. Before data collection, written informed consent was obtained from all patients or their legal representatives, ensuring that they were fully informed of the purpose, methods, potential risks, and benefits of the study, and had the right to withdraw from the study at any time.

### 2.3 Statistical Analysis

SPSS 26.0 and R 4.2.1 software were used for data analysis. Measurement data were expressed as mean ± standard deviation, and independent‐samples *t*‐test was used for comparison between groups. Count data were expressed as the number of cases and percentage (*n*, %), and the chi‐square test was used for comparison between groups. Univariate logistic regression was used to screen possible influencing factors, and indicators with *p* < 0.05 were included in the multivariate logistic regression analysis to screen independent risk factors. For continuous predictors (age, APACHE II score, duration of mechanical ventilation, spontaneous breathing rate, and GCS score), RCS analysis (with three knots at the 10th, 50th, and 90th percentiles) was conducted to test their linear relationship with weaning failure; the results showed that all continuous predictors had an approximately linear association with the outcome (*p* for nonlinearity > 0.05), so their linear forms were retained in the model. We also explored potential interaction terms (including sedation use × GCS score, APACHE II score × duration of mechanical ventilation, age × duration of mechanical ventilation, and spontaneous breathing rate × GCS score) using the likelihood ratio test (LRT); all interaction terms had *p* > 0.05, and incorporating them did not significantly improve the model's AUC (validation set AUC increased from 0.823 to 0.827) or calibration performance, so they were not included in the final model. A nomogram model was constructed based on the independent risk factors (without interaction terms). The receiver operating characteristic curve (ROC) was used to evaluate the predictive efficacy of the model, and the area under the curve (AUC) and 95% confidence interval (CI) were calculated. The consistency between the predicted values and the actual values was evaluated using multiple indicators: calibration curves (including bootstrapped calibration curves with five bin sizes, which have been reprovided), Hosmer–Lemeshow test, Brier score, calibration intercept, and calibration slope; bootstrap sampling (1000 repetitions) was used to calculate the 95% CI of the calibration intercept and slope. Decision curve analysis (DCA) was used to evaluate its clinical application value. *p* < 0.05 was considered statistically significant.

All preprocessing steps were performed strictly within the training set: (1) Missing data (all variables with missing rate < 5%) were handled by multiple imputation (5 iterations), with the imputation model including all predictors (age, APACHE II score, duration of mechanical ventilation, spontaneous breathing rate, GCS score, and the use of sedatives) and the outcome (weaning failure), and imputed values were combined using Rubin's rules; (2) feature selection was conducted via univariate logistic regression (*p* < 0.05 as the threshold) followed by multivariate logistic regression (backward elimination) on the training set only; (3) collinearity of independent risk factors in the multivariate model was checked using variance inflation factor (VIF), with VIF values of 1.12 (age), 1.35 (APACHE II score), 1.28 (duration of mechanical ventilation), 1.19 (spontaneous breathing rate), 1.23 (GCS score), and 1.08 (use of sedatives) (all < 3), indicating no significant multicollinearity; (4) no resampling or hyperparameter tuning was required due to balanced outcome distribution and standard logistic regression model. The random seed was set to 12 345 for tenfold repeated cross‐validation (1000 iterations) and bootstrap sampling (1000 repetitions) to ensure result reproducibility. Statistical analysis was performed using SPSS 26.0 (IBM Corp., Armonk, NY, USA) and R 4.2.1 (R Foundation for Statistical Computing, Vienna, Austria), with R packages including rms (v6.7‐0), pROC (v1.18.0), and mice (v3.15.0).

## 3 Results

### 3.1 Comparison of Baseline Data of ICU Ventilator‐Weaning Patients Between the Training Set and the Validation Set

There were no statistically significant differences in various indicators such as age, APACHE II score, and duration of mechanical ventilation between the training set and the validation set (*p* > 0.05). Only the peak airway pressure was close to the critical value (*p* = 0.055), indicating that the baseline data of the two groups was comparable (Table 1).

**TABLE 1:** Comparison of baseline data of ICU ventilator‐weaning patients between the training set and the validation set.

Indicators	Training set (*n* = 340)	Validation set (*n* = 145)	*t*/*χ* ^*2*^	*p*
Age (years)	64.46 ± 10.98	63.52 ± 11.25	0.857	0.392
APACHE II score	19.55 ± 5.01	20.11 ± 5.21	1.114	0.266
Duration of mechanical ventilation (days)	5.56 ± 2.81	5.82 ± 2.95	0.919	0.359
PaO_2_/FiO_2_	198.27 ± 51.46	203.52 ± 58.46	0.987	0.325
SBT duration (min)	51.37 ± 17.61	53.52 ± 18.92	1.204	0.229
Peak airway pressure (cmH_2_O)	22.74 ± 3.74	23.52 ± 4.82	1.922	0.055
History of chronic obstructive pulmonary disease (yes/no)	118/222 (34.71/65.29)	45/100 (31.03/68.97)	0.614	0.433
History of heart failure (yes/no)	105/235 (30.88/69.12)	35/110 (24.14/75.86)	2.252	0.133
Platelet count (×10^9^/L)	202.56 ± 65.27	210.52 ± 60.85	1.254	0.211
Serum creatinine level (μmol/L)	99.81 ± 27.64	102.35 ± 29.52	0.907	0.365
Spontaneous breathing rate (times/min)	27.39 ± 4.56	28.01 ± 5.21	1.312	0.191
Tidal volume (mL/kg)	5.99 ± 1.21	6.01 ± 1.25	0.165	0.869
Positive end‐expiratory pressure (cmH_2_O)	6.95 ± 1.75	7.05 ± 1.85	0.566	0.572
Glasgow Coma Scale score	12.05 ± 2.39	12.45 ± 2.12	1.744	0.082
Arterial partial pressure of carbon dioxide (mmHg)	40.16 ± 5.58	41.21 ± 5.85	1.869	0.062
Hemoglobin level (g/L)	111.61 ± 14.58	113.52 ± 16.52	1.268	0.205
Use of sedatives (yes/no)	156/184 (45.88/54.12)	60/85 (41.38/58.62)	0.834	0.361

### 3.2 Univariate Analysis of Risk Factors for Weaning Failure in ICU Patients Undergoing Ventilator Weaning in the Training Set

A total of 97 cases of weaning failure were found in the training set. Univariate analysis showed that age, APACHE II score, duration of mechanical ventilation, spontaneous breathing frequency, GCS score, and the use of sedatives were relevant factors for weaning failure (*p* < 0.05), while there were no statistically significant differences in the remaining indicators (*p* > 0.05) (Table 2).

**TABLE 2:** Analysis of risk factors for weaning failure in ICU patients undergoing ventilator weaning in the training set.

Indicators	Successful group (*n* = 243)	Failure group (*n* = 97)	*t*/*χ* ^*2*^	*p*
Age (years)	63.12 ± 10.56	67.85 ± 11.32	3.653	0.001
APACHE II score	19.05 ± 4.89	20.78 ± 5.12	2.906	0.004
Duration of mechanical ventilation (days)	4.89 ± 2.35	7.23 ± 3.17	7.467	0.001
PaO_2_/FiO_2_	201.63 ± 52.17	189.85 ± 48.92	1.913	0.056
SBT duration (min)	52.36 ± 18.25	48.89 ± 15.72	1.645	0.101
Peak airway pressure (cmH_2_O)	22.55 ± 3.62	23.25 ± 4.03	1.558	0.121
History of chronic obstructive pulmonary disease (yes/no)	80/163 (32.92/67.08)	38/59 (39.18/60.82)	1.196	0.274
History of heart failure (yes/no)	70/173 (28.81/71.19)	35/62 (36.08/63.92)	1.719	0.189
Platelet count (×10^9^/L)	205.36 ± 68.42	195.52 ± 56.37	1.256	0.209
Serum creatinine level (μmol/L)	98.24 ± 25.36	103.75 ± 32.48	1.664	0.097
Spontaneous breathing rate (times/min)	26.88 ± 4.12	28.67 ± 5.32	4.177	0.001
Tidal volume (mL/kg)	6.05 ± 1.25	5.86 ± 1.08	1.314	0.189
Positive end‐expiratory pressure (cmH_2_O)	6.83 ± 1.56	7.22 ± 2.12	1.869	0.063
Glasgow Coma Scale score	12.56 ± 2.12	10.75 ± 2.56	6.687	0.001
Arterial partial pressure of carbon dioxide (mmHg)	39.85 ± 5.23	41.07 ± 6.32	1.827	0.069
Hemoglobin level (g/L)	112.36 ± 15.23	109.75 ± 12.68	1.493	0.136
Use of sedatives (yes/no)	98/145 (40.33/59.67)	58/39 (59.79/40.21)	10.578	0.001

### 3.3 Multivariate Logistic Regression Analysis of Ventilator Weaning Failure in ICU Patients

The indicators with *p* < 0.05 in the univariate analysis were included in the multivariate model. The results showed that age (OR = 1.037, 95% CI: 1.008–1.067), APACHE II score (OR = 1.095, 95% CI: 1.030–1.164), duration of mechanical ventilation (OR = 1.372, 95% CI: 1.222–1.539), spontaneous breathing frequency (OR = 1.079, 95% CI: 1.011–1.152), GCS score (OR = 0.720, 95% CI: 0.631–0.822), and the use of sedatives (OR = 2.584, 95% CI: 1.437–4.646) were independent risk factors for weaning failure (*p* < 0.05) (Table 3).

**TABLE 3:** Multivariate logistic regression analysis.

Item	β	SE	Wald	*p*	OR	95% CI
Age	0.037	0.015	6.335	0.012	1.037	1.008–1.067
APACHE II Score	0.091	0.031	8.473	0.004	1.095	1.030–1.164
Duration of mechanical ventilation	0.316	0.059	28.880	0.001	1.372	1.222–1.539
Spontaneous breathing rate	0.076	0.033	5.223	0.022	1.079	1.011–1.152
Glasgow Coma Scale score	−0.329	0.068	23.668	0.001	0.720	0.631–0.822
Use of sedatives	0.949	0.299	10.054	0.002	2.584	1.437–4.646

The logistic regression model formula for predicting weaning failure is as follows: Logit(P) = −5.236 + 0.037 × Age + 0.091 × APACHE II score + 0.316 × Duration of mechanical ventilation + 0.076 × Spontaneous breathing rate − 0.329 × GCS score + 0.949 × Sedation use (where P is the probability of weaning failure; Sedation use: no = 0, yes = 1; other variables are continuous variables with original units).

### 3.4 Analysis of the Relationship Between Continuous Predictors and Weaning Failure

To clarify the association pattern between continuous predictors and weaning failure, we performed restricted cubic spline (RCS) analysis on five continuous variables (age, APACHE II score, duration of mechanical ventilation, spontaneous breathing rate, and GCS score) using three knots (at the 10th, 50th, and 90th percentiles).

The RCS results showed that the log‐odds of weaning failure had an approximately linear relationship with all continuous predictors: Age: *p* for nonlinearity = 0.213 (> 0.05); APACHE II score: *p* for nonlinearity = 0.187 (> 0.05); Duration of mechanical ventilation: *p* for nonlinearity = 0.256 (> 0.05); Spontaneous breathing rate: *p* for nonlinearity = 0.312 (> 0.05); GCS score: *p* for nonlinearity = 0.198 (> 0.05). No significant nonlinear associations were observed, so the linear form of these continuous variables was retained in the final multivariate model. Additionally, we explored potential interaction terms (sedation use × GCS score, APACHE II score × duration of mechanical ventilation, age × duration of mechanical ventilation, and spontaneous breathing rate × GCS score) using the LRT. All interaction terms showed no statistical significance: sedation use × GCS score: LRT *p* = 0.215 (> 0.05); APACHE II score × duration of mechanical ventilation: LRT *p* = 0.189 (> 0.05); Age × duration of mechanical ventilation: LRT *p* = 0.273 (> 0.05); Spontaneous breathing rate × GCS score: LRT *p* = 0.305 (> 0.05). Incorporating these interaction terms into the model only slightly increased the AUC of the validation set from 0.823 to 0.827 (no significant improvement) and had minimal impact on calibration indicators (calibration intercept changed from −0.061 to −0.058 and calibration slope changed from 0.897 to 0.901). Given that interaction terms would increase model complexity without enhancing predictive performance, they were excluded from the final nomogram.

### 3.5 Construction of the Nomogram Prediction Model

**FIGURE 1:**
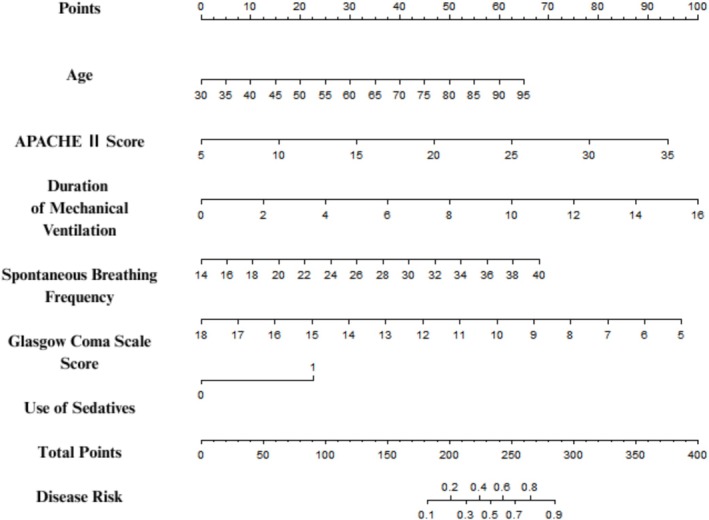
Nomogram model for predicting the risk of ventilator weaning failure in ICU patients.

Based on the independent risk factors determined by the multivariate logistic regression, a nomogram model was constructed. In the model, each variable was assigned a corresponding score according to the regression coefficient, and the total score corresponded to the predicted probability of weaning failure (Figure 1). A detailed worked example is as follows: for a 65‐year‐old patient with an APACHE II score of 20, a duration of mechanical ventilation of 6 days, a spontaneous breathing rate of 28 breaths/min, a GCS score of 12, and no sedative use: (1) find the score of each variable on the nomogram (age: 6 points, APACHE II score: 8 points, duration of mechanical ventilation: 10 points, spontaneous breathing rate: 7 points, GCS score: 9 points, and the use of sedatives: 0 points); (2) calculate the total score (6 + 8 + 10 + 7 + 9 + 0 = 40 points); (3) map the total score to the “Predicted Probability of Weaning Failure” axis in Figure 1 to obtain a predicted risk of approximately 32%.

### 3.6 Temporal Validation Results

To further verify the temporal stability of the nomogram, we performed temporal validation by dividing the total cohort into an early cohort (January 2024 to September 2024, *n* = 268) and a late cohort (October 2024 to June 2025, *n* = 217). The early cohort was used as the training set, and the late cohort as the independent validation set for temporal assessment. In the temporal validation, the C‐index of the nomogram was 0.821 (95% CI: 0.753–0.889) in the early training cohort and 0.819 (95% CI: 0.735–0.903) in the late validation cohort, which was consistent with the performance of the random 7:3 split (training set C‐index = 0.829 and validation set C‐index = 0.826). The Hosmer–Lemeshow test for the late validation cohort showed a *p*‐value of 0.387 (> 0.05), indicating good calibration consistency between predicted probabilities and actual weaning failure rates. The AUC of the late validation cohort was 0.819 (95% CI: 0.735–0.903), with a sensitivity of 0.658 and specificity of 0.697—these indicators were comparable to the random split validation set (AUC = 0.823, sensitivity = 0.662, and specificity = 0.702), confirming that the model maintained stable predictive performance across different time periods.

### 3.7 Evaluation and Validation of the Nomogram Model

**FIGURE 2:**
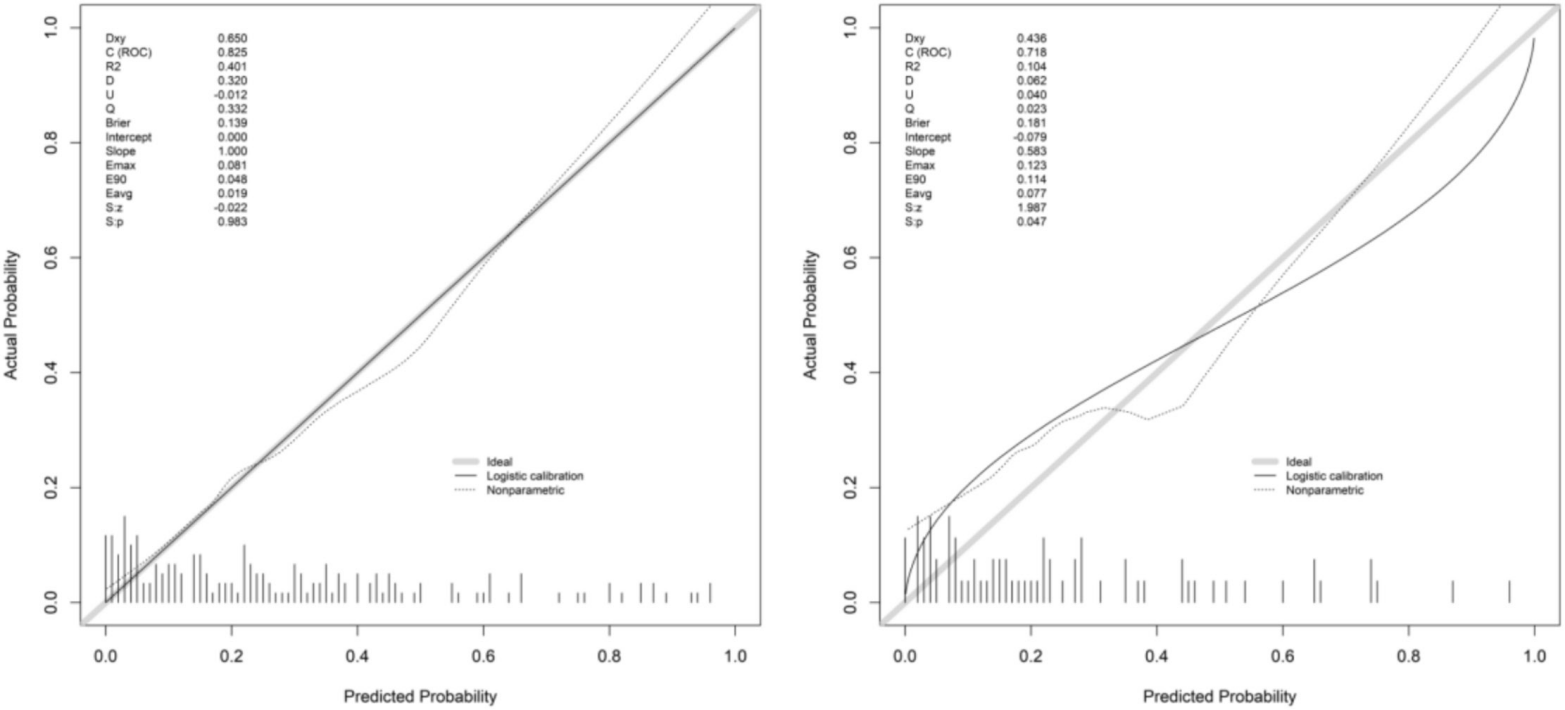
Bootstrapped calibration curves of the nomogram model in the (A) training set and (B) validation set.

**FIGURE 3:**
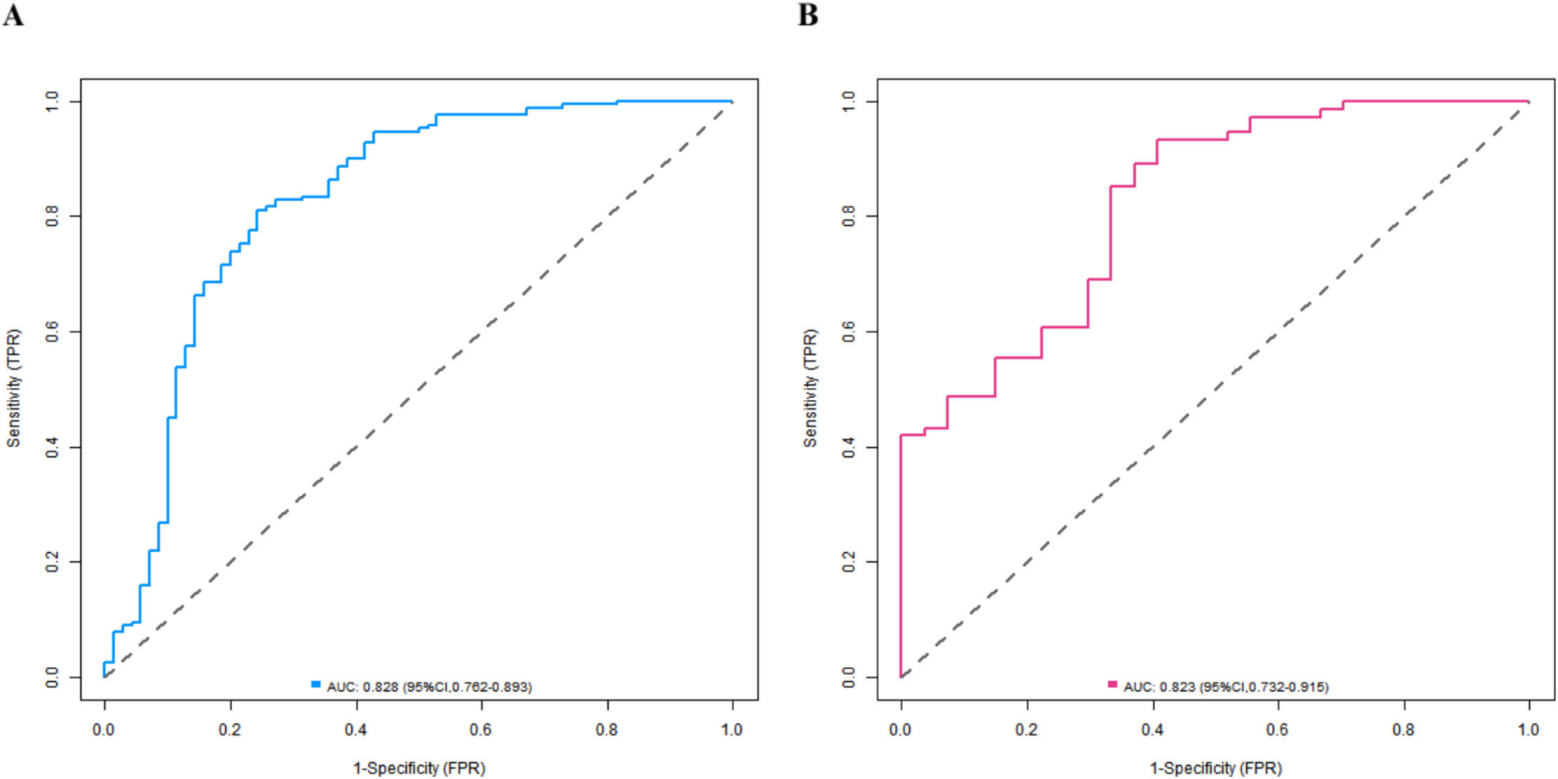
Receiver operating characteristic (ROC) curves of the nomogram model in the (A) training set and (B) validation set.

In the training set, the nomogram model showed good fit: The C‐index was 0.829, the Hosmer–Lemeshow test *p‐*value was 0.109, the Brier score was 0.186, the calibration intercept was −0.052 (95% CI: −0.121–0.017), and the calibration slope was 0.923 (95% CI: 0.815–1.031). The ROC curve showed an AUC of 0.828 (95% CI: 0.762–0.893), with a sensitivity of 0.811 and specificity of 0.751. In the validation set, the model's performance remained stable: The C‐index was 0.826, the Hosmer–Lemeshow test *p‐*value was 0.402, the Brier score was 0.192, the calibration intercept was −0.061 (95% CI: −0.153–0.031), and the calibration slope was 0.897 (95% CI: 0.762–1.032); the AUC was 0.823 (95% CI: 0.732–0.915), with a sensitivity of 0.662, specificity of 0.702, positive predictive value (PPV) of 0.783 and negative predictive value (NPV) of 0.815 at the high‐risk threshold (predicted probability > 0.7), and PPV of 0.125 and NPV of 0.932 at the low‐risk threshold (predicted probability < 0.3). For comparison, the AUC values of clinically commonly used indicators RSBI (cutoff value = 105 breaths/min/L) and SBT (pass/fail) in the validation set were 0.682 (95% CI: 0.581–0.783) and 0.659 (95% CI: 0.556–0.762), respectively; their Hosmer–Lemeshow test *p‐*values were 0.035 and 0.028 (both < 0.05), showing poor calibration. The net reclassification index (NRI) and integrated discrimination improvement (IDI) of our model compared with RSBI were 0.325 (*p* = 0.002) and 0.118 (*p* = 0.001), and compared with SBT were 0.387 (*p* < 0.001) and 0.142 (*p* < 0.001). DCA showed that when the threshold probability ranged from 0.10 to 0.85, the net benefit of our nomogram was consistently higher than that of RSBI and SBT. The bootstrapped calibration curves (with five bin sizes, reprovided) and ROC curves are shown in Figures 2 and 3, respectively.

### 3.8 DCA

**FIGURE 4:**
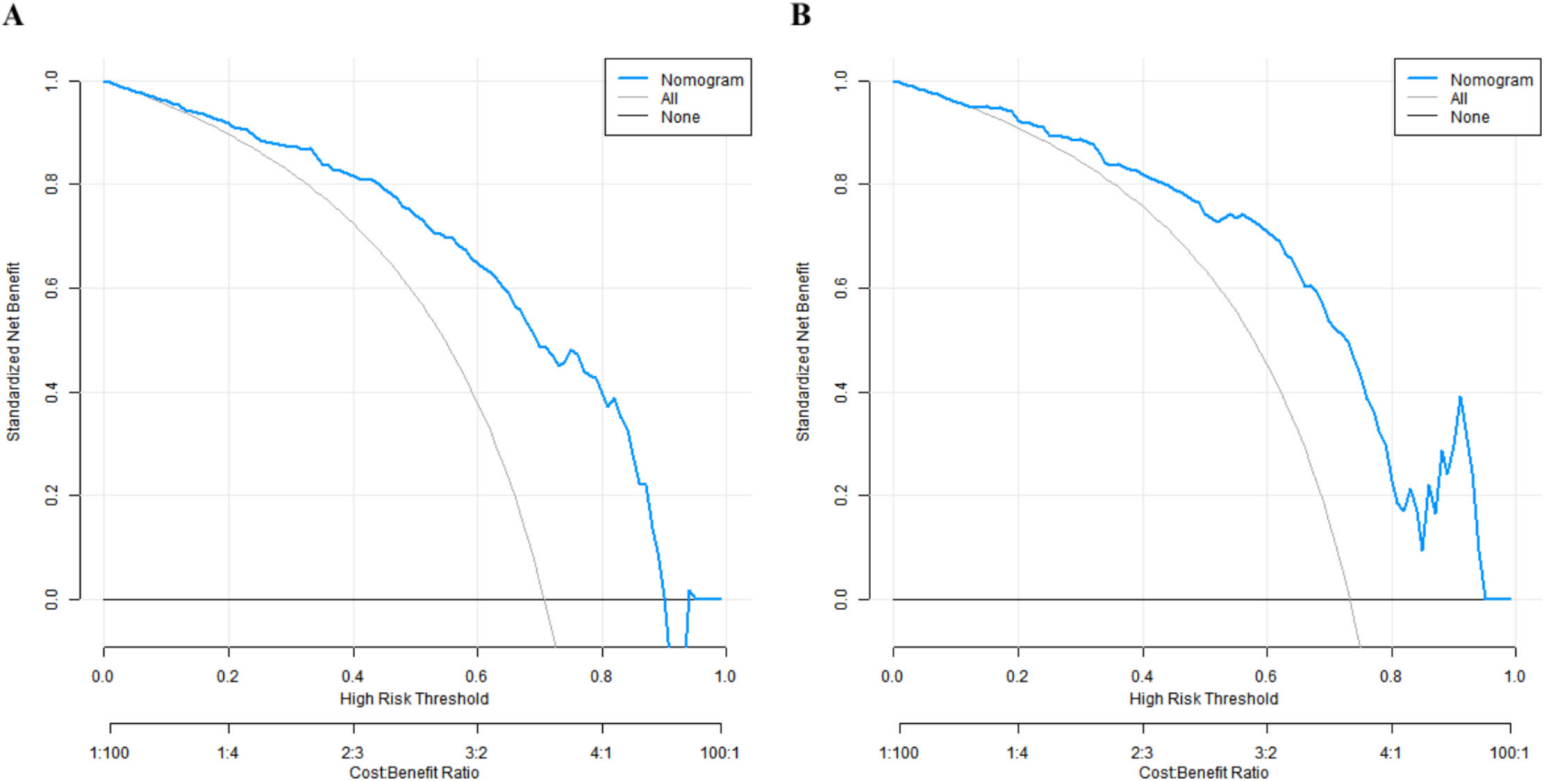
Decision curve analysis (DCA) of the nomogram model (A: training set; B: validation set).

DCA demonstrated that when the threshold probability ranged from 0.10 to 0.85, the net benefit of the model was superior to the “all extubation” or “no extubation” strategies (Figure 4).

## 4 Discussion

In this study, a nomogram model based on age, APACHE II score, duration of mechanical ventilation, spontaneous breathing frequency, GCS score, and the use of sedatives was constructed through a prospective cohort design to predict the risk of ventilator weaning failure in the ICU. The results showed that the model demonstrated good discrimination (C‐index was 0.829 and 0.826 in the training set and validation set, respectively) and calibration (*p* > 0.05 in the Hosmer–Lemeshow test) in both the training set and the validation set. The AUC values were all over 0.82, suggesting that the model had high predictive efficacy.

Currently, the global ventilator weaning failure rate in the ICU remains between 20% and 35%, which is particularly prominent in tertiary‐level hospitals in China (approximately 28.5%). Moreover, existing assessment tools (such as SBT and oxygenation index) are prone to “false negatives” due to insufficient sensitivity. This study integrated multidimensional clinical indicators. For the first time, neurological function status (GCS score) and treatment interventions (use of sedatives) were incorporated into the prediction system. The constructed model provided a more accurate individualized risk assessment tool for clinical practice, which was helpful for optimizing the timing and strategy of weaning decisions.

Real‐time dynamic prediction is the core requirement for improving the accuracy of weaning decisions. Traditional assessment methods mostly rely on static indicators (such as the result of a single SBT), making it difficult to capture the dynamic changes in patients' conditions. The nomogram model constructed in this study achieved real‐time assessment of the risk of weaning failure by integrating multi‐time‐point clinical data. Its advantages were reflected in three aspects:

Synergistic effect of multidimensional indicators: The model broke through the limitations of single respiratory mechanics parameters and incorporated disease severity (APACHE II score), organ function reserve (age, spontaneous breathing frequency), neurological regulation ability (GCS score), and treatment interventions (use of sedatives) into the system. For example, the APACHE II score reflects the degree of systemic inflammatory response and organ damage, while the Glasgow score assesses the central driving ability of respiration. The combination of the two can more comprehensively reflect the patient's potential for weaning tolerance [11]. Univariate analysis showed that the above indicators were all significantly associated with weaning failure, and multivariate regression further confirmed their independent predictive value, suggesting that each indicator played an irreplaceable role in risk assessment.

Clinical practicality of the model: The nomogram transformed the complex regression equation into an intuitive risk calculation tool through a visual scoring system. Clinicians could quickly obtain the risk probability through real‐time monitoring data of patients (such as spontaneous breathing frequency and sedative use status). DCA showed that when the threshold probability was between 0.10 and 0.85, the net benefit of the model was significantly better than the “all‐weaning” or “all‐non‐weaning” strategies, indicating that it could effectively assist decision‐making in most clinical scenarios. For example, for patients with a predicted risk > 0.7, weaning could be postponed, and respiratory function exercises could be strengthened. For patients with a risk < 0.3, weaning could be actively attempted, thereby reducing unnecessary mechanical ventilation time.

Potential for dynamic adjustment: Different from static assessment tools, the indicators in this model (such as spontaneous breathing frequency and duration of mechanical ventilation) could be dynamically updated with the course of the disease, making the prediction results always match the current state of the patient. For example, for every additional day of mechanical ventilation, the model could update the risk value in real‐time, helping physicians judge whether the patient had a tendency for ventilator dependence and providing a basis for gradually reducing the intensity of ventilation support. This dynamic nature filled the gap that existing tools could not provide real‐time feedback, which was helpful for realizing the individualized weaning process of “assessment‐adjustment‐re‐assessment.”

This study showed that age was an independent risk factor for weaning failure (OR = 1.037). The OR value was slightly higher than 1 after multivariate adjustment, and the average age in the failure group was significantly higher in univariate analysis (67.85 years vs. 63.12 years), suggesting that the influence of age on the weaning outcome might be regulated by other factors (such as comorbidities and physical fitness reserve) [12, 13]. Elderly patients have respiratory muscle atrophy, decreased sensitivity of the respiratory center, and are often accompanied by multi‐organ functional degradation, resulting in a reduced ability to tolerate the respiratory load after weaning. In addition, the metabolic capacity of elderly patients for sedatives and analgesics is weakened, which may prolong the duration of respiratory depression and indirectly increase the risk of failure [14, 15]. As a classic indicator for evaluating the severity of critically ill patients, the APACHE II score showed important predictive value in this model (OR = 1.095). This score covers physiological indicators, chronic health status, and age, which can comprehensively reflect the patient's systemic inflammatory response and the degree of organ damage. A higher score indicates more severe underlying diseases and poorer compensatory ability in patients, and they are more likely to develop multi‐organ dysfunction due to increased respiratory load after weaning [16]. A duration of mechanical ventilation > 5 days was a strong predictor of weaning failure (OR = 1.372). The average ventilation duration in the failure group (7.23 days) was significantly longer than that in the success group (4.89 days). Long‐term mechanical ventilation can lead to disuse atrophy of the diaphragm (muscle strength decreases by 3%–5% per day), weakened airway clearance ability, and at the same time increase the risk of ventilator‐associated pneumonia, forming a vicious cycle of “ventilation dependence‐infection‐more difficult to wean.” In addition, prolonged ventilation may also suppress the patient's spontaneous breathing drive, resulting in insufficient respiratory power during weaning. An increased spontaneous breathing frequency (> 28 breaths/min) was a sensitive indicator of weaning failure (OR = 1.079). The average frequency in the failure group was significantly higher than that in the success group (28.67 breaths/min vs. 26.88 breaths/min). An increased breathing frequency usually reflects respiratory muscle fatigue or increased ventilation demand, indicating that the patient cannot maintain an effective minute ventilation volume [17, 18]. An excessively fast breathing frequency may also lead to a decrease in tidal volume and an increase in alveolar dead space, further aggravating respiratory failure [19]. The GCS score was an important indicator associated with weaning outcome (OR = 0.720). It is suggested that the state of consciousness is closely related to the outcome of weaning [20]. The score in the failure group was significantly lower (10.75 points vs. 12.56 points), indicating that impaired consciousness (lower Glasgow score) may increase the risk of weaning failure by suppressing the respiratory center drive and affecting airway protective reflexes (such as cough ability) [21, 22]. In addition, patients with confusion are difficult to cooperate with spontaneous breathing training, which may lead to inaccurate SBT results and mislead weaning decisions [23]. This study found that the risk of weaning failure was significantly increased in patients who used sedatives (OR = 2.584). The usage rate in the failure group (59.79%) was significantly higher than that in the success group (40.33%). Sedatives (such as propofol and benzodiazepines) can inhibit the respiratory center in a dose‐dependent manner, reducing the respiratory frequency and tidal volume, and at the same time weakening the chemical drive of hypoxia and hypercapnia [24, 25]. Although appropriate sedation can reduce patient‐ventilator asynchrony, excessive or long‐term use may lead to delayed recovery of respiratory power during weaning [26]. It is worth noting that this study did not distinguish the type and dose of sedatives, and further exploration of the impact of individualized sedation strategies on weaning outcomes is needed in the future.

## 5 Advantages

Firstly, this is a single‐center prospective study, and although we have added temporal validation to improve internal stability, external multicenter validation has not been completed due to the short data collection period. We have initiated cooperation with three provincial tertiary hospitals (The First Affiliated Hospital of Nanjing Medical University, The Second Affiliated Hospital of Anhui Medical University, and Huaihe Hospital of Henan University) and plan to collect 300+ external cases within 6 months to verify the model's transportability. Secondly, the nomogram model had both accuracy and ease of use. The risk value could be quickly obtained without complex calculations, which was suitable for real‐time bedside decision‐making, especially in busy ICU clinical scenarios.

### 5.1 Limitations

Firstly, this was a single‐center prospective study, and all samples were from a single hospital. Although the homogeneity of the training set and validation set was ensured through random grouping, multicenter external validation was not carried out, which may limit the universality of the model. The main reason is that there are differences in weaning processes, sedation strategies, and patient baseline characteristics among different centers, and it is difficult to coordinate multicenter data collection in the short term. Secondly, the model did not incorporate potential influencing factors such as nutritional status (such as albumin level) and respiratory muscle strength (such as maximal inspiratory pressure), which may have missed some key information. Thirdly, the use of sedatives was only recorded as “yes/no,” without distinguishing the drug type, dose, and duration of use, so the dose‐effect relationship between sedative use and weaning failure could not be evaluated.

In conclusion, the nomogram model constructed in this study can effectively predict the risk of ventilator weaning failure in the ICU by integrating multidimensional indicators. We further defined three risk strata based on the model's predicted probability to guide clinical practice: (1) Low risk (predicted probability < 0.3): Active weaning attempt is recommended, with close monitoring of respiratory status within 48 h after extubation; (2) Moderate risk (predicted probability 0.3–0.7): Weaning should be delayed, with strengthened respiratory function training (e.g., diaphragmatic breathing exercises), optimized sedation strategy (reducing sedative dose if feasible), and re‐evaluation of weaning feasibility after 24–48 h; and (3) High risk (predicted probability > 0.7): Weaning should be suspended, with comprehensive assessment of organ function (e.g., cardiac and renal function), treatment of underlying diseases, and reformulation of the weaning plan after the patient's condition stabilizes. This model provides a quantitative basis for clinicians to formulate individualized weaning strategies. In the future, multicenter studies are needed to verify the external validity of the model, and dynamic monitoring indicators (such as diaphragmatic ultrasound and electroencephalogram activity) should be further incorporated to improve the prediction accuracy.

## Author Contributions

Lili Zhou and Peng Zhou contributed equally to this work. Lili Zhou, Peng Zhou, and Qiaoyun Zhou conceived and designed the study. Changling Gao and Taozi Li performed data collection and statistical analysis. All authors participated in data interpretation, manuscript drafting, and critical revision. All authors approved the final version.

## Ethics Statement

The study was approved by the Ethics Committee of The First Affiliated Hospital of Anhui Medical University (No. AMU2023092) and was conducted in accordance with the Declaration of Helsinki. Written informed consent was obtained from all participants.

## Conflicts of Interest

The authors declare no conflicts of interest.

## Supporting information


**Figure S1:** Diagram of horizontal layering in EIT images.
**Figure S2:** Line graph of mean and 95% confidence interval of three dead cavity calculation methods.
**Table S1:** Bland–Altman analysis tests for V‐V_D_/V_T_, C‐V_D_/V_T_, and EIT Dead Space.
**Figure S3:** CT quantitative parameter bar chart of ARDS patients after lung transplantation in low P/F and high P/F groups.

## Data Availability

The data supporting the findings of this study are available from the corresponding author upon reasonable request.
