# Supplementary material for: Real‐Time Predictive Analysis of ICU Ventilator Weaning Failure: A Prospective Validation Study
Source: Clin Respir J. 2025 Nov 4;19(11):e70136. doi: 10.1111/crj.70136 (PMC12585916; doi:10.1111/crj.70136)
Supplement: Supplementary file 1 — Figure S1: Diagram of horizontal layering in EIT images. Figure S2: Line graph of mean and 95% confidence interval of three dead cavity calculation methods. Table S1: Bland–Altman analysis tests for V‐VD/VT, C‐VD/VT, and EIT Dead Space. Figure S3: CT quantitative parameter bar chart of ARDS patients after lung transplantation in low P/F and high P/F groups. [file CRJ-19-e70136-s001.docx]

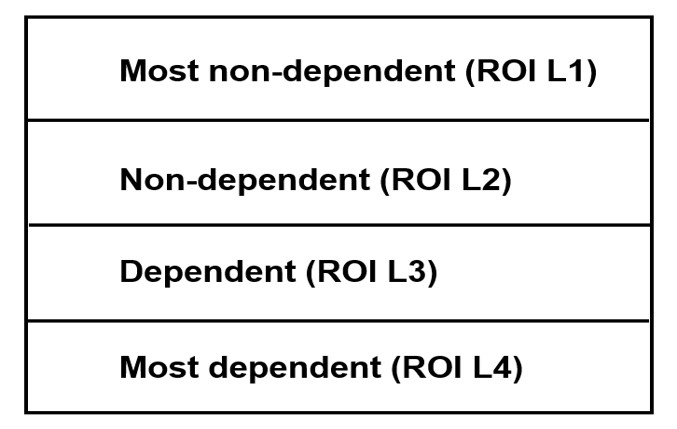


FigureS1. Diagram of horizontal layering in EIT images.


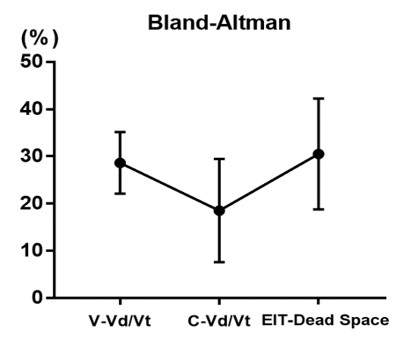


FigureS2. Line graph of mean and 95% confidence interval of three dead cavity calculation methods.


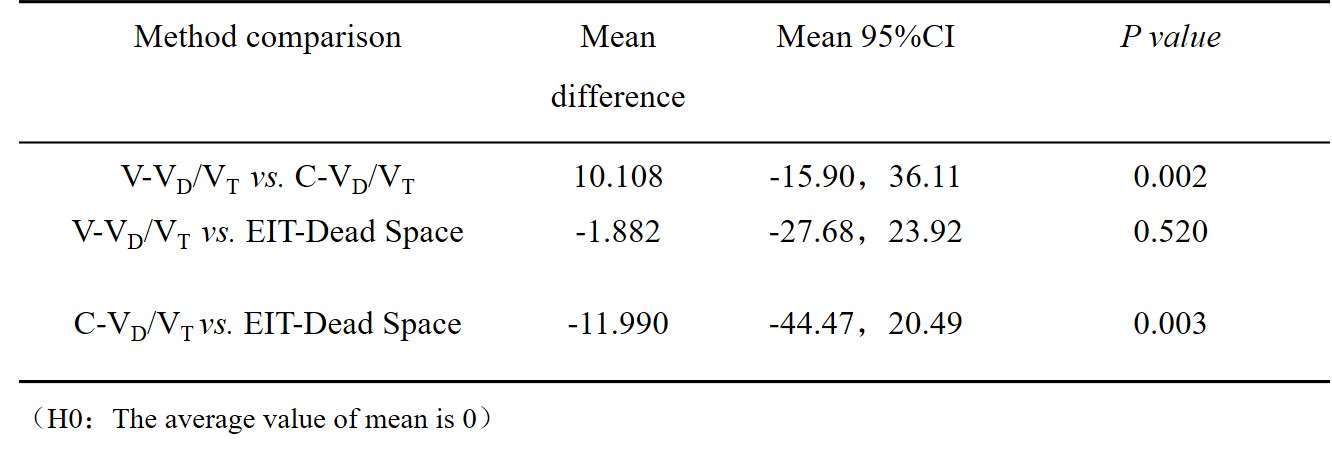


TableS1. Bland-Altman analysis tests for V-V_D_/V_T_, C-V_D_/V_T_, and EIT Dead Space.


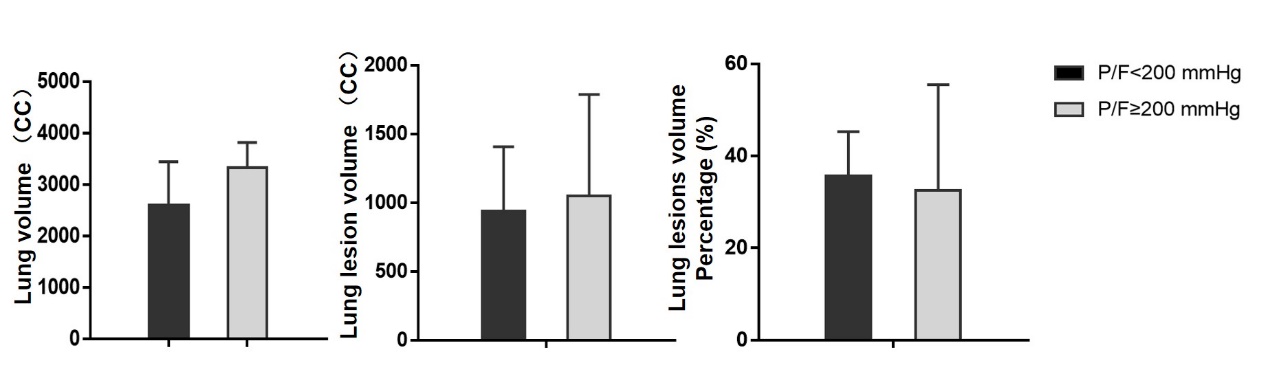
 FigureS3. CT quantitative parameter bar chart of ARDS patients after lung transplantation in low P/F and high P/F groups.
